# The gut bacteria across life stages in the synanthropic fly *Chrysomya megacephala*

**DOI:** 10.1186/s12866-018-1272-y

**Published:** 2018-10-11

**Authors:** Xiaoyun Wang, Qiao Gao, Wanqiang Wang, Xiaoping Wang, Chaoliang Lei, Fen Zhu

**Affiliations:** 1 Hubei International Scientific and Technological Cooperation Base of Waste Conversion by Insects, Huazhong Agricultural University, Wuhan, 430070 China; 2 Hubei Insect Resources Utilization and Sustainable Pest Management Key Laboratory, Huazhong Agricultural University, Wuhan, 430070 China

**Keywords:** *Chrysomya megacephala*, Developmental stage, Microbiota, 16S rDNA sequencing

## Abstract

**Background:**

Gut bacteria are closely associated with host. *Chrysomya megacephala*, as a vector and resource insect, can transmit various pathogenic bacteria and consume manure to produce biofertilizer and larva biomass. However, the gut bacteria composition and abundance of *C. megacephala* remain unclear.

**Results:**

Illumina MiSeq platform was used to compare composition of gut bacterial community in eggs, 1-day-old larvae, 5-day-old larvae, pupae, adult females and males by sequencing with variation in V4 region of 16S ribosomal DNA gene. In total, 928 operational taxonomic units (OTUs) were obtained. These OTUs were annotated into 19 phyla, 42 classes, 77 orders, 153 families and 289 genera. More than 0.5% abundance of 32 OTU core genera were found across all life stages. At class level, *Alphaproteobacteria*, *Bacilli*, *Bacteroidia*, *Betaproteobacteria*, *Flavobacteriia* and *Gammaproteobacteria* were the most abundant in *C. megacephala*. Eight species were identified to have significantly different abundance between 1-d-larvae and 5-day-larvae and took 28.95% of shared species between these two groups. Sex-specific bacterial species were identified that *Faecalibacterium prausnitzii* was merely present in females, while *Rhodococcus fascians* was merely present in males.

**Conclusion:**

Gut bacteria of *C. megacephala* varied across life stages. The composition and community structure of the bacterial community differed from young larvae to mature larvae, while that were similar in adult females and males. These data will provide an overall view of bacterial community across life stages in *C. megacephala* with attention on manure associated and pathogenic bacteria.

**Electronic supplementary material:**

The online version of this article (10.1186/s12866-018-1272-y) contains supplementary material, which is available to authorized users.

## Background

Environmentally acceptable treatments are indispensable to overcome environmental concerns raising up due to increasing manure production by livestock industry [1]. Fly larvae have been successfully used to reduce mass of animal manure and yield biofertilizer and nutrient-rich larval products [2, 3]. The larvae of *Chrysomya megacephala* can consume different types of manure [4, 5] and the manure transformation system by *C. megacephala* larvae are capable of reducing the waste in a short period of time while providing maggot biomass and bio-fertilizer simultaneously [6]. Improvement and environmental safety control of this system will help speed up manure processing, improve fertilizer efficiency, further protect the environment and control health risk.

Insect gut bacteria are closely associated with feed digestion [7], especially for specific food types, such as blood-sucking bugs and wood/soil-consuming termites and herbivorous insects [8–11]. Gut bacteria mining of herbivorous insects with typical cultural method and metagenome sequencing was conducted to identify beneficial microbes that possess cellulase activity [12, 13]. Parallel saprophagous fly *Musca domestica* larval gut was reported as a digestion chamber altered antibiotic resistome of swine manure other than merely digested manure [14]. In the manure transformation system by *C. megacephala*, larvae play similar role in digestion [6], which might also act this way*.* Therefore, mining of gut bacteria from larvae might help provide digestion promoting and candidate environmentally beneficial bacteria. However, the composition of gut bacteria in *C. megacephala* has not been reported yet.

Manure transformation system by *C. megacephala* also raise health concerns because *C. megacephala* are vectors for transmitting microorganisms. Many bacteria attach to the external surface of *C. megacephala* [15], especially on adults [16]. Notably, *C. megacephala* can load 11–12 times greater bacteria than that of housefly *Musca domestica* and some of the bacteria were human pathogenic enteric bacteria, i.e. *Salmonella* sp., *Shigella* sp. [17]. Several experiments have been conducted to identify the pathogenic and non- pathogenic bacteria that were carried by *C. megacephala* [18–20]. In Sinop of Brazil, *Burkholderia* sp. had the largest part of the identified pathogenic bacteria in *C. megacephala* [18]. Moreover, *C. megacephala* was recently found to be a vector for *Wohlfahrtiimonas chitiniclastica* which cause infections of human [19]. In Grahamstown of South Africa, *Bacillus pumilus* were none-pathogenic and abundant in *C. megacephala*, representing 80.37% of the total colonies [20]. Therefore, bacteria on external surface of *C. megacphala* are relevant to external environments. However, the presence of pathogenic bacteria in gut are unknown.

In addition, gut bacteria are associated with development, reproduction, resistance and management of host insect. For example, in dung beetle, *Onthophagus gazelle*, symbionts play a role in mediating its normal development [21]. *Enterobacter cloacae*, *Providencia stuartii*, *Pusillimonas* sp., *Pedobacter heparinus*, and *Lysinibacillus sphaericus* were isolated from brood ball of the dung beetle, *Onthophagus taurus* and found to play a role in nutrition supplement [22]. Female gut harboured more abundant bacteria than male in the red turpentine beetle *Dendroctonus valens* which might be connected with reproduction [23]. Gut symbiont enhances insecticide resistance in the oriental fruit fly, *Bactrocera dorsalis* (Hendel) [24]. Mining gut bacteria across all life stages of host insects provide an overall view of bacterial variations of host insects and also propose potential biocontrol techniques against pest [25] and benefit host insect breeding [26].

In this study, interior/intestinal bacteria of across life stages in manure-feeding of *C. megacephala* were sequenced vastly by 16S rDNA in V4 region from eggs, 1-day-old larvae, 5-day-old larvae, pupae, females and males to generally illuminate the gut bacteria composition. Comparative analysis of gut bacteria between 1-day-old larvae and 5-day-old larvae was specially conducted to understand the changes of gut bacteria in early and late stages during manure transformation. Adult female and male were also compared to address sexual differences of gut bacteria with an eye on pathogenic bacteria. These results would provide valuable bacterial pool of *C. megacephala* and would further contribute in improving larval manure transformation, increasing egg production and developing adult management techniques.

## Methods

### Insect rearing and sample collection

Laboratory *C. megacephala* was provided by the Hubei International Scientific and Technological Cooperation Base of Waste Conversion by Insects (Wuhan, China). Adults of *C. megacephala* were reared in mesh cages (35 × 35 × 35 cm) with the water solution of sugar and the cages were kept in a rearing room at 25 ± 3 °C under a 13:11 h light: dark photoperiod.

Swine manure was taken from the swine breeding farm of Huazhong Agricultural University (Wuhan, China) for manure consuming experiments by *C. megacephala*. Adults were reared as mentioned above for egg production. Eggs were firstly collected with a swine manure gauze bag by putting into cages for 4 h and then eggs were separated from the gauze. A pile of egg mass was collected into1.5 mL sterile centrifuge tubes and then washed with 75% alcohol following deionized water. Eggs separated in deionized water and thirty of them were counted and collected into a new 1.5 mL sterile centrifuge tubes by pipetting with tips. The water in the tubes were removed and then the eggs were stored at − 80 °C. Later, most of the remaining eggs were loaded on manure in proportion of 1.5 g eggs per kilo gram manure. Ten 1-day-old larvae were sampled 1 day after the egg oviposition. As time went on, 5-day-old larvae, 3-day-old pupae, 4-day-old females and males were starved for 2 h, washed as eggs and then dissected in phosphate Buffer solution (PBS) for alimentary tracts or content. Finally, thirty eggs, ten of 1-day-old larvae, 5 alimentary tracts of 5-day-old larvae, 5 content (Tissues were sampled by eliminating puparium with fine tweezers) of 2-day-old pupae, 5 alimentary tracts of 4-day-old females and 5 alimentary tracts of 4-day-old males were sampled and stored at − 80 °C before use. The samples were abbreviated as Eggs, 1-d-Larvae, 5-d-Larvae, Pupae, Female and Male, respectively. For different developmental stages, per three individual replicates of the same generation was conducted. Totally, 18 samples were used for DNA extraction.

### Genomic DNA preparation, PCR and sequencing

Genomic DNA was extracted from all sampled tubes using a TIANamp Genomic DNA Kit (TIANGEN Biotech: DP304, Beijing, China). Sequencing for the bacterial variable V4 regions of the 16S rDNA gene was performed by BGI-Tech (BGI Tech Solutions Co., Ltd., Wuhan) on the Illumina MiSeq platform. Each PCR reaction contained 30 ng of genomic DNA from an individual sample, as well as V4 Dual-index Fusion PCR Primer Cocktail and NEB Phusion High-Fidelity PCR Master Mix (New England Biolabs, Inc., US). The primers are 515F (5’-GTGCCAGCMGCCGCGGTAA-3′) and 806R (5’-GGACTACHVGGGTWTCTAAT3’) and an approximately 270 bp fragment on the V4 region of the 16S rDNA gene of the bacteria were obtained as described [27]. Thermocycling conditions included an annealing temperature of 56 °C and a total of 30 cycles. The PCR products were purified with AmpureXP beads (AGENCOURT, Beckman Coulter, Inc., US) to remove primer dimers and unused PCR reagents. The final library was quantitated in two ways: Firstly, by determining the average molecule length using the Agilent 2100 bioanalyzer instrument (Agilent DNA 1000 Reagents, Agilent Inc., USA), and secondly, by quantifying the library with real-time quantitative PCR (QPCR) (EvaGreen™, EGFIE LLC, USA). The qualified libraries were paired-end sequenced on a MiSeq System, using the sequencing strategy PE250 (PE251 + 8 + 8 + 251) (MiSeq Reagent Kit v3, Illumina Inc., US).

### Bioinformatics and statistical analysis

Raw sequences of all samples were processed as previously described to obtain clean data [28]. Six groups of bacterial communities were found across the life stages of *C. megacephala* with three replicates: Eggs, 1-d-Larvae, 5-d-Larvae, Pupae, Female and Male. All samples were then applied for Tag-generation by FLASH and Operational taxonomic units (OTUs) cluster analysis using USEARCH [29, 30]. OTU classification, alignment of the representative sequence of each OTU, chimaera removal, taxonomic assignment and alpha and beta diversity analyses were performed with QIIME (macQIIME 1.7) [31]. Differential analyses of abundant microbial communities between groups were conducted using Metastats (http://metastats.cbcb.umd.edu/) at the levels of i.e. phylum, class, order, family, genus and species [32]. The obtained *P*-value by a Benjamini-Hochberg false discovery rate correction (function ‘p.adjust’ in the stats package of R(v3.1.1)) was adjusted within Metastats. Software R(v3.1.1) was used to analyse data and figures were made by R(v3.1.1) along with GraphPad 5.0 and the OmicShare tools, a free online platform for data analysis (http://www.omicshare.com/tools/).

## Results

### Gut bacterial diversity

**Fig. 1:**
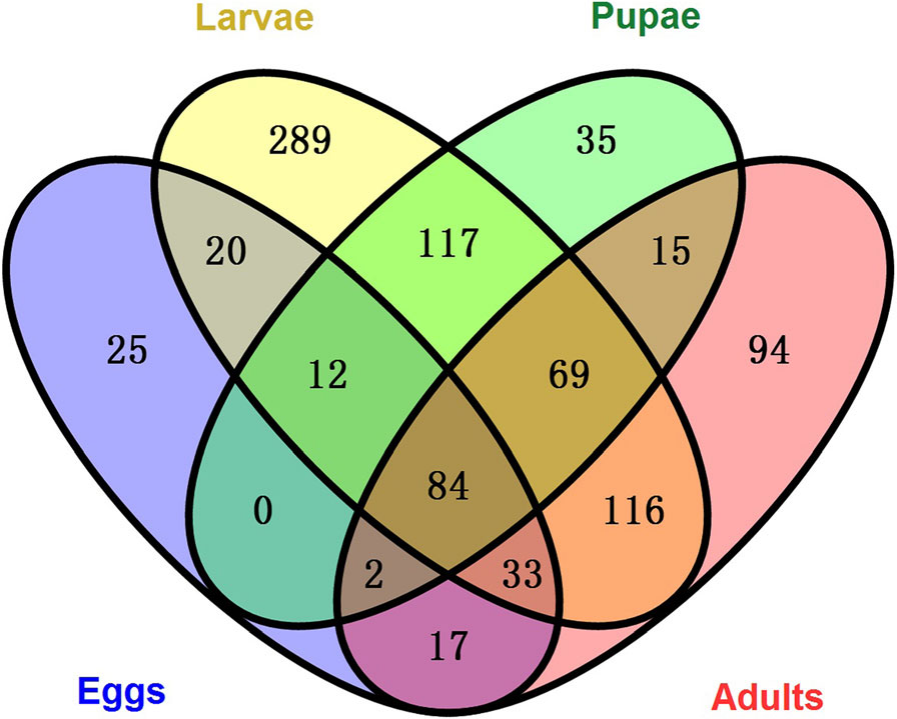
Venn diagram of OTU distribution across *C. megacephala* life stages. Numbers within compartments indicate OTU counts of according to mathematical sets

**Fig. 2:**
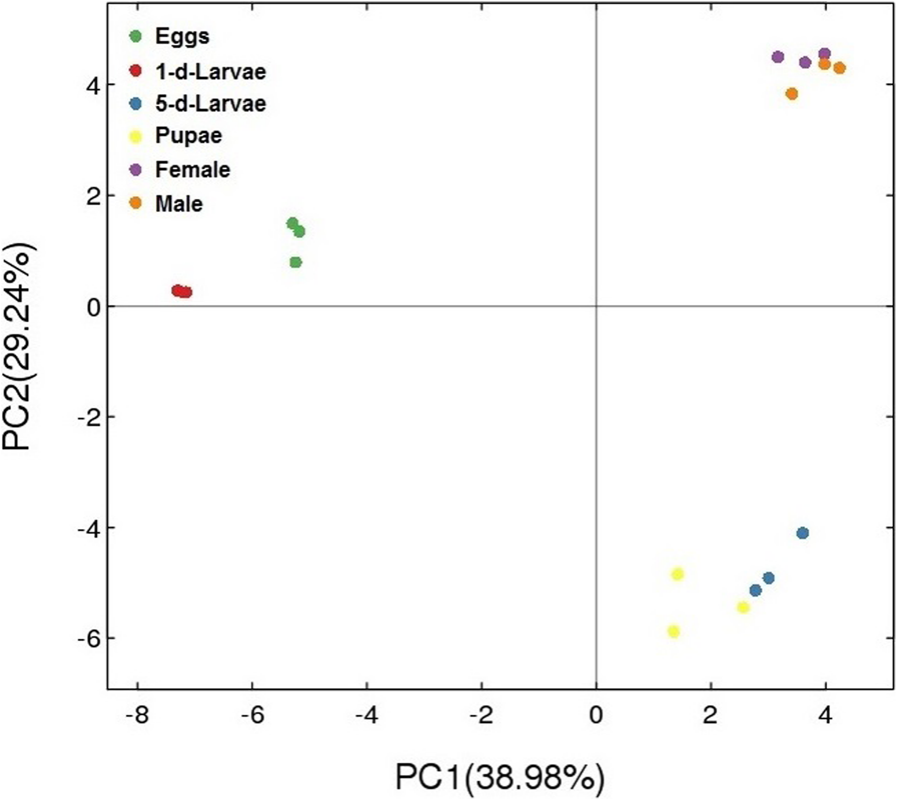
Principal component analysis based on OTUs. X-axis, 1st principal component; Y-axis, 2nd principal component. Numbers in parentheses represent the contributions of the principal components to differences among samples. Dots represents individual samples, and different colours represent different groups. This plot was made by Package “ade4” of software (v3.1.1)

A total of 584,493 raw reads were obtained and 563,245 clean reads were generated from *C. megacephala* (Additional file 1: Table S1). Nine hundred and twenty-eight operational taxonomic units (OTUs) were generated from all samples. These OTUs were annotated into 19 phyla, 42 classes, 77 orders, 153 families and 289 genera (Additional file 2: Table S2), of which 84 OTUs were shared across all life stages (Fig. 1). Sufficient sequencing data were obtained based on the plateaued rarefraction curves of obvious species (Additional file 3: Figure S1). Based on the OTU abundance information (97% similarity), the relative abundance of each OTU in each sample were calculated, and the PCA (Principal component analysis) of OTU was done with the relative abundance value (Fig. 2). Coordinate dots of female and male samples were closely located. The distance comparisons to origin indicated that their OTU compositions were similar (student’s *t* test, *p* = 0.87). Likewise, the relative abundance of 1-d-larvae were close to eggs but not 5-d-larvae based on different vector location. Gut bacteria of larval samples were more diversified than that of other samples which were derived from five diversity estimators in Table 1. Higher value of Observed species (sobs), Chao, Ace, Simpson’s index and lower Shannon’s index in 1-d-larvae and 5-d-larvae groups suggested that gut bacteria from larval guts were more diverse than those from other life stages.

**Table 1:** Bacterial alpha diversity of *C. megacephala* in different life stages based on the 16S rDNA amplicon

Sample	Sobs	Chao	Ace	Shannon	Simpson
Eggs	137 ± 22.65	158.05 ± 14.38	162.88 ± 14.72	2.49 ± 0.40	0.17 ± 0.06
1-d-Larvae	289.5 ± 10.79	368.38 ± 41.48	367.76 ± 27.40	3.03 ± 0.08	0.09 ± 0.01
5-d-Larvae	302.25 ± 38.73	374.76 ± 34.85	388.73 ± 38.42	2.47 ± 0.46	0.22 ± 0.06
Pupae	250 ± 40.50	297.00 ± 53.33	320.10 ± 39.20	2.53 ± 0.45	0.22 ± 0.08
Female	203.75 ± 9.29	269.69 ± 19.89	301.08 ± 35.10	2.48 ± 0.08	0.19 ± 0.01
Male	145 ± 5.13	191.50 ± 21.19	228.49 ± 11.69	2.40 ± 0.15	0.17 ± 0.03

### Taxonomic view of gut Bacteria across life stages

**Fig. 3:**
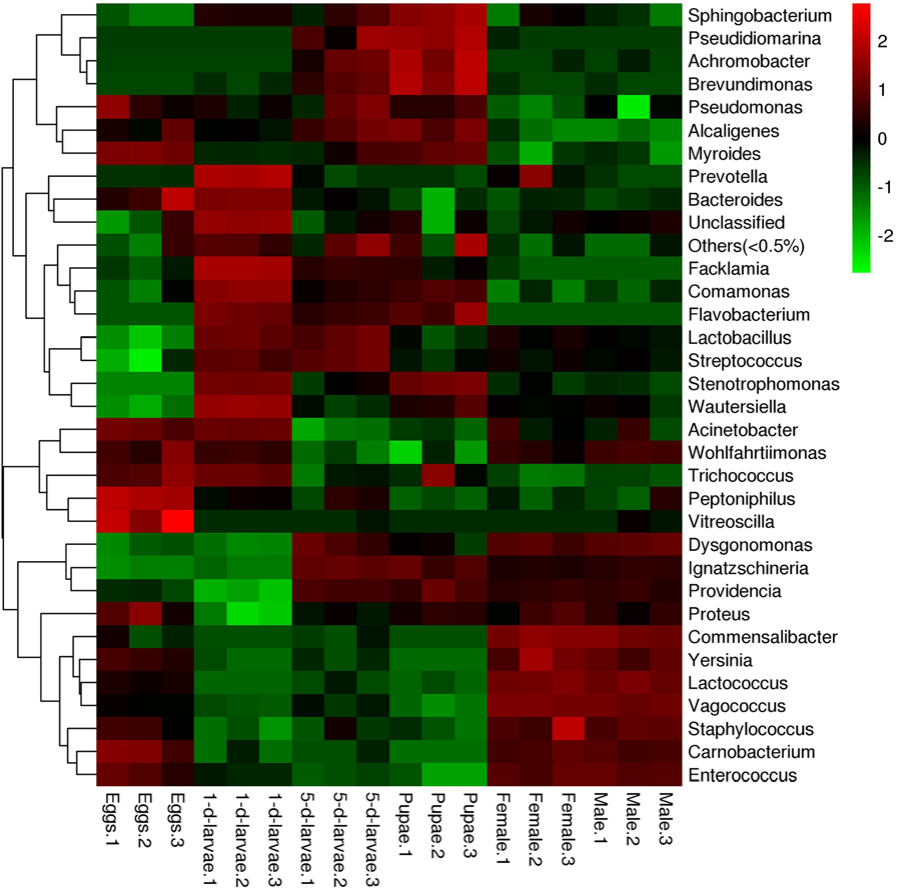
Heatmap of the relative abundance of bacterial communities at the genus level across *C. megacephala* life stages. Heatmaps was generated suing the OmicShare tools, a free online platform for data analysis (http://www.omicshare.com/tools/). The species of which abundance is less than 0.5% in all samples were classified into ‘others’. The species was not classified into database were marked by ‘unclassified’

**Fig. 4:**
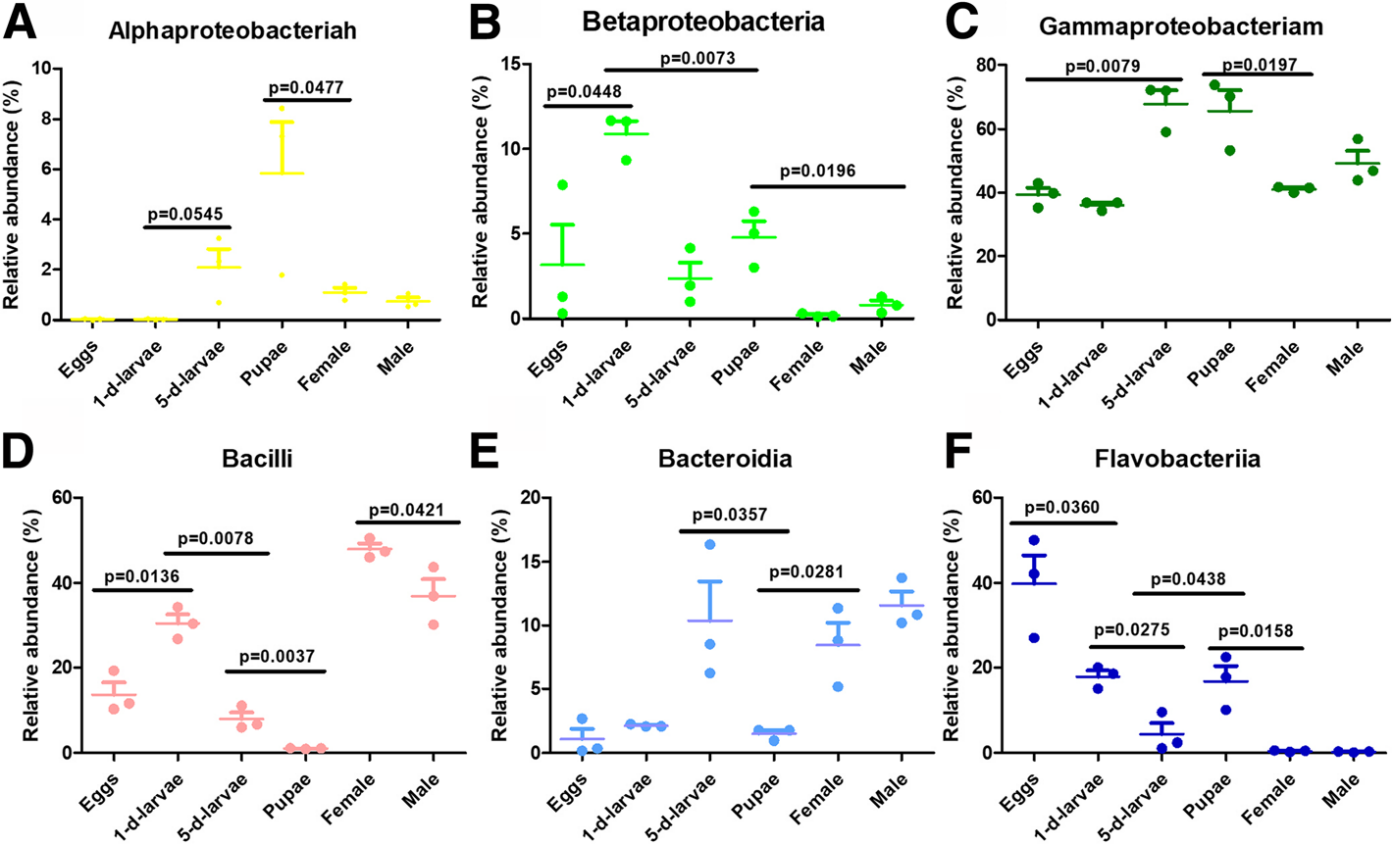
The taxonomic composition distribution in samples of Class-level. **a**: *Alphaproteobacteria*; **b**: *Betaproteobacteria*; **c**: *Gammaproteobacteria*; **d**: *Bacilli*; **e**: *Bacteroidia*; **f**: *Flavobacteriia.* The average ratio of each bacteria class in samples across life stages is directly displayed. Y-axis indicated the relative abundance of microbial communities between samples. Points showed three individual values, longer bars in each column indicated mean values while shorter bars indicate SE value. Metastats (http://metastats.cbcb.umd.edu/) and R(v3.1.1) were used to determine which taxonomic groups were significantly different between groups of samples with the obtained *p*-value by a Benjamini-Hochberg false discovery rate correction (function ‘p.adjust’ in the stats package of R(v3.1.1)

The distribution of gut bacteria communities at the genus level was viewed by heatmap (Fig. 3). Samples from each group were mostly clustered together which indicated a good repeatability. The involved genera in Fig. 3 were provided with counts of OTUs (Additional file 4: Table S3). Figure 4 presented the relative abundances of different bacterial classes. Six known bacterial classes represented the majority. These were *Alphaproteobacteria*, *Bacilli*, *Bacteroidia*, *Betaproteobacteria*, *Flavobacteriia* and *Gammaproteobacteria*. Among these classes, *Gammaproteobacteria* and *Bacilli* had the highest number of reads. Overall, relative bacterial class composition varied with the development of *C. megacephala.* Moreover, successive *C. megacephala* metamorphosis or insect states shared similar gut bacteria compositions at the class level (Fig. 4).

### Comparative analysis between manure-consuming larval samples

Larval stage is manure consuming period in *C. megacephala* [4, 5]. 1-d-Larvae and 5-d-Larvae groups represented the gut bacteria from manure in early and late period of transformation. In total, eight species were identified to have significantly different abundance in above mentioned larval groups and they took 28.95% of shared species between these two groups (Table 2). Seven of the identified species decreased from 1-d-Larvae to 5-d-Larvae groups, only one species *Pseudoclavibacter bifida* increased (Table 2).

**Table 2:** Eight differential abundance of bacterial species between 1-d-Larvae and 5-d-Larvae Groups of *C. megacephala*

Species	1-d-Larvae		5-d-Larvae		*p*-value
	Mean	SE	Mean	SE	
*Arcobacter cryaerophilus*	0.150963	0.028609	0.002329	0.001165	0.014(−)
*Bacteroides coprosuis*	0.484238	0.032372	0.022096	0.000914	0.0035(−)
*Bulleidia p-1630-c5*	0.024685	0.007027	0	0	0.038094(−)
*Escherichia coli*	0.02612	0.001494	0.013998	0.002089	0.021(−)
*Eubacterium biforme*	0.040341	0.008873	0.00548	0.003131	0.031094(−)
*Faecalibacterium prausnitzii*	0.020832	0.003285	0.00119	0.00119	0.007(−)
*Flavobacterium gelidilacus*	0.363321	0.058491	0.070145	0.012969	0.0175(−)
*Prevotella stercorea*	0.063915	0.011388	0	0	0.0105(−)
*Pseudoclavibacter bifida*	0.002632	0.002632	0.060935	0.018523	0.045094(+)
*Pseudomonas alcaligenes*	0.140686	0.023227	0.030898	0.027479	0.048594(−)
*Ruminococcus gnavus*	0.023545	0.006094	0.00119	0.00119	0.034594(−)

Note: Metastats (http://metastats.cbcb.umd.edu/) and R (v3.1.1) are used to determine which taxonomic groups were significantly different between groups of samples. We adjusted the obtained P-value by a Benjamini-Hochberg false discovery rate correction (function ‘p.adjust’ in the stats package of R(v3.1.1)). (−) indicated a significant decrease in abundance from 1-d-Larvae to 5-d-Larvae; while (+) indicated reversely

### Comparative analysis between adult samples

Adult stage is dominant pathogen transmission period [15] and control strategies might draw inspiration from sex difference so that comparative analysis between adult samples were addressed. Distribution of gut bacterial communities were similar based on the close location in the PCA plot and the Metastats indicated that most of dominating bacteria had no significant difference between female and male at different taxonomic levels. However, sex-specific bacterial species were identified. *Faecalibacterium prausnitzii* was merely present in females (*p* = 0.03607), while *Rhodococcus fascians* was merely present in males (*p* = 0.01075) (Additional file 5: Table S4).

## Discussion

### Comparisons of gut Bacteria with other insects

Results in this experiment indicated that the composition of gut bacteria in *C. megacephala* were relatively more diversified than some reported dipteral insects. The obtained sequences were generated to 928 OTUs from *C. megacephala* at 97% sequencing identity (Fig. 1, Table 1 and Additional file 1: Table S1). Only 122, 197 OTUs were generated from the gut of *Drosophila melanogaster* and *B. dorsalis* respectively by a similar sequencing method [25, 33]. However, the number of OTUs were generated more than twice as much as *C. megacephala* in the gut of polyphagous tomato fruit borer, *Helicoverpa armigera* (Hübner), indicating that diet and environment might affect gut bacteria of host insect [34, 35]. The result that bacterial diversity of *C. megacephala* varied along with eggs, larvae, pupae and adults were accordance with that of other holometabolic dipteral insects, such as *B. dorsalis* and *Musca domestica* [25, 36].

Moreover, dominating gut bacteria present similarities and differences among *C. megacephala* and *M. domestica.* Comparative discussions were conducted because *M. domestica* is a one of the most important relative species of *C. megacephala* and they are always found concurrently in nutrient substance, such as food waste, manure and carrion [37]. Generally, in phylum level, gut bacteria were similar in *M. domestica* and *C. megacephala. Proteobacteria*, *Firmicutes* and *Bacteroidetes* were proved to be predominant phylum in gut bacteria of *C. megacephala* (Fig. 4). Similar conclusion was also drawn in housefly *M. domestica* [36, 38], which might result from a similar ecological niche [16]. Gut bacteria in these flies showed some difference at detailed taxonomic level, In dairy manure consuming *M. domestica*, *Bacilli*, *Clostridia*, *Actinobacteria*, *Flavobacteria*, and *Proteobacteria* were the most abundant classes [39] and *Bacilli*, *Flavobacteria*, *Proteobacteria* were also included as abundant classes in swine manure consuming *C. megacephala* (Fig. 4). The wheat bran consuming *M. domestica* larvae also contained *Ignatzschineria* as a dominated genus in gut bacteria [40], indicating that polyphagous synanthropic fly larvae might share some of core harbored bacteria because of overlap of food range since *M. domestica* are also manure-consuming [14].

### Gut bacteria of larva *C. megacephala*

The relative abundance of gut bacteria in 1-d-larvae differed much in 5-d-larve (Table 2 and Additional file 6: Table S5). The changes of bacterial community in young and old larvae might result from the manure feeding activity of larvae by manure transformation and bacterial digestion [36]. Fasting in the mature larvae might also play a role in this difference since mature larvae would experience a wandering stage to empty gut autonomously [41]. In some higher animals, gut bacteria *Coprobacillus* and *Ruminococcus* exhibited decrease in response to fasting [42]. Strikingly, pathogenic genera *Wohlfahrtiimonas*, swine manure associated *Brevundimonas diminuta* and *Flavobacterium gelidilacus* decreased significantly (Table 2) [26, 36], which reduced health concerns. While, *Pseudoclavibacter bifida* increased significantly (Table 2) and it was an infection-associated organism that may cause chronic obstructive pulmonary disease [43]. The increase of this bacterium might result from the accumulation of undigested bacteria which need further attention, since it might colonize from larvae to newly emerged adults like other pathogens, i.e. *Providencia* spp. [40].

*C. megacephala* shared some manure associated bacteria with manure microbiota. Pig slurry predominantly comprised members of the *Bacteroidetes*, *Firmicutes* and *Proteobacteria* phyla [44], which shared some of gut bacteria in larva *C. megacephala*. In the identified bacterial species of *C. megacephala*, *Enterococcus* took a certain proportion in gut. Moreover, manure associated microbiota might have attractive effect to insects. For example, *Rhizobium*, *Devosia* and *Brevundimonas* in horse manure had the most stimulation to oviposit effect of the stable fly *Stomoxys calcitrans* [45], indicating that these shared bacteria between gut and manure might also promote egg production of *C. megacephala* to enlarge the manure transformation system. Therefore, shared bacteria in manure and *C. megacephala* gut also need further researches.

### Gut bacteria of adult *C. megacephala*

Pathogenic bacteria were observed as minority in adult gut based on taxonomic results. Among them, *Escherichia coli* and *Streptococcus luteciae* might have pathogenicity in inflammatory bowel disease [46] which should be monitored. At genus level, *Wohlfahrtiimonas sp.* and *Ignatzschineria* are opportunistic pathogens [47, 48], which should also be kept under observation.

In comparative view on female and male, no significant difference was detected in predominate bacteria between sex (Figs. 2 and 3). However, thirty-three genera were only detected in female, and 12 were only detected in male (Additional file 7: Table S6). Some bacteria species might have a sex-specific representation such as *Faecalibacterium prausnitzii* and *Rhodococcus fascians* (Additional file 4: Table S3 and Additional file 5: Table S4). *Faecalibacterium prausnitzii* is a beneficial gut microbe to human and have anti- inflammatory role [49, 50]. *Rhodococcus fascians* is interestingly identified to be a plant phytopathogenic actinobacterium which causes leafy galls and other plant distortions that result in economically significant losses to nurseries producing ornamental plants [51]. Further research in their function might promote egg production and provide control target of sex-specific attractant.

## Conclusion

This study used 16S ribosomal DNA sequencing to clarify the intracorporeal bacteria of *C. megacephala* across life stages. These results suggested that gut bacteria of *C. megacephala* varied across life stages and *Alphaproteobacteria*, *Bacilli*, *Bacteroidia*, *Betaproteobacteria*, *Flavobacteriia* and *Gammaproteobacteria* were the most abundant classes in *C. megacephala*. The relative abundance of the bacterial community differed from young larvae to mature larvae, while that were similar from female to male.

## Additional files

Additional file 1: **Table S1.** Samples and their sequencing data processing. (DOCX 15 kb)

Additional file 2: **Table S2.** OTU taxonomy of all samples. (XLSX 35 kb)

Additional file 3: **Figure S1.** Rarefaction curve based on OTUs. Mothur (v1.31.2) were used to calculate indices for rareaction curve based on observed species values. (DOCX 130 kb)

Additional file 4: **Table S3.** Numbers OTU that belong to core genera. (XLSX 9 kb)

Additional file 5: **Table S4.** Comparative abundance of bacterial species between female and male of *C. megacephala. (DOCX 17 kb)*

Additional file 6: **Table S5.** Relative abundance of 1-d-Larvae and 5-d-Larvae at genus level. (XLSX 21 kb)

Additional file 7: **Table S6.** Relative abundance of female and male at genus level. (XLSX 18 kb)

## Abbreviations

16S rDNA: 16S ribosomal DNA gene
OTU: Operational taxonomic units
PBS: Phosphate Buffer solution
PCA: Principal component analysis
PCR: Polymerase chain reaction
QIIME: Quantitative insights into microbial ecology
qPCR: Real-time PCR

## References

[1] Bidart C, Froehling M, Schultmann F (2014). Livestock manure and crop residue for energy generation: macro-assessment at a national scale. Renew Sust Energ Rev.

[2] Li Z, Yang D, Huang M, Hu X, Shen J, Zhao Z (2012). *Chrysomya megacephala* (Fabricius) larvae: a new biodiesel resource. Appl Energ..

[3] Cickova H, Newton GL, Lacy RC, Kozanek M (2015). The use of fly larvae for organic waste treatment. Waste Manag.

[4] Gabre RM, Adham FK, Chi H (2005). Life table of *Chrysomya megacephala* (Fabricius)(Diptera: Calliphoridae). Acta Oecol.

[5] Hu Y, Yuan X, Zhu F, Lei C (2010). Development time and size-related traits in the oriental blowfly, *Chrysomya megacephala* along a latitudinal gradient from China. J Therm Biol.

[6] Yang S, Liu Z (2014). Pilot-scale biodegradation of swine manure via *Chrysomya megacephala* (Fabricius) for biodiesel production. Appl Energ.

[7] Hanning I, Diaz-Sanchez S. The functionality of the gastrointestinal microbiome in non-human animals. Microbiome. 2015;3. 10.1186/s40168-015-0113-6. 10.1186/s40168-015-0113-6 PMC4640220 26552373

[8] Chu CC, Spencer JL, Curzi MJ, Zavala JA, Seufferheld MJ (2013). Gut bacteria facilitate adaptation to crop rotation in the western corn rootworm. Proc Natl Acad Sci U S A.

[9] Rossmassler K, Dietrich C, Thompson C, Mikaelyan A, Nonoh JO, Scheffrahn RH, et al. Metagenomic analysis of the microbiota in the highly compartmented hindguts of six wood- or soil-feeding higher termites. Microbiome. 2015;3. 10.1186/s40168-015-0118-1. 10.1186/s40168-015-0118-1 PMC4660790 26607965

[10] Wadakatsumata A, Zurek L, Nalyanya G, Roelofs WL, Zhang A, Schal C (2015). Gut bacteria mediate aggregation in the german cockroach. Proc Natl Acad Sci U S A.

[11] Benjamino J, Graf J (2016). Characterization of the core and caste-specific microbiota in the termite, *Reticulitermes flavipes*. Front Microbiol.

[12] Handique Gautam, Phukan Amrita, Bhattacharyya Badal, Baruah Abu Adil Lutful Haque, Rahman Syed Wasifur, Baruah Rajen (2017). Characterization of cellulose degrading bacteria from the larval gut of the white grub beetleLepidiota mansueta(Coleoptera: Scarabaeidae). Archives of Insect Biochemistry and Physiology.

[13] Duarte S, Duarte M, Borges PAV, Nunes L (2017). Dietary-driven variation effects on the symbiotic flagellate protist communities of the subterranean termite *Reticulitermes grassei* Clement. J Appl Entomol.

[14] Wang Hang, Sangwan Naseer, Li Hong-Yi, Su Jian-Qiang, Oyang Wei-Yin, Zhang Zhi-Jian, Gilbert Jack A, Zhu Yong-Guan, Ping Fan, Zhang Han-Luo (2016). The antibiotic resistome of swine manure is significantly altered by association with the Musca domestica larvae gut microbiome. The ISME Journal.

[15] Sukontason KL, Bunchu N, Methanitikorn R, Chaiwong T, Kuntalue B, Sukontason K (2006). Ultrastructure of adhesive device in fly in families calliphoridae, muscidae and sarcophagidae, and their implication as mechanical carriers of pathogens. Parasitol Res.

[16] Sukontason KL, Bunchoo M, Khantawa B, Piangjai S, Rongsriyam Y, Sukontason K (2007). Comparison between *Musca domestica* and *Chrysomya megacephala* as carriers of bacteria in northern Thailand. Se Asian J Trop Med.

[17] Chaiwong T, Srivoramas T, Sueabsamran P, Sukontason K, Sanford M, Sukontason K (2014). The blow fly, *Chrysomya megacephala*, and the house fly, *Musca domestica*, as mechanical vectors of pathogenic bacteria in Northeast Thailand. Trop Biomed.

[18] Carneiro JS, Pires EM, Nogueira RM, Shiomi HF, Soares MA, Oliveira MA (2014). Bacteria carried by *Chrysomya megacephala* (Fabricius, 1794) (Diptera: Calliphoridae) in Sinop, Mato Grosso, Brazil. Sci Electron Arch.

[19] Schrottner P, Rudolph WW, Damme U, Lotz C, Jacobs E, Gunzer F (2017). *Wohlfahrtiimonas chitiniclastica*: current insights into an emerging human pathogen. Epidemiol Infect.

[20] Brits D, Brooks M, Villet MH (2016). Diversity of bacteria isolated from the flies *Musca domestica* (Muscidae) and *Chrysomya megacephala* (Calliphoridae) with emphasis on vectored pathogens. Afr Entomol.

[21] Schwab DB, Riggs HE, Newton ILG, Moczek AP (2016). Developmental and ecological benefits of the maternally transmitted microbiota in a dung beetle. Am Nat.

[22] Estes Anne M., Hearn David J., Snell-Rood Emilie C., Feindler Michele, Feeser Karla, Abebe Tselotie, Dunning Hotopp Julie C., Moczek Armin P. (2013). Brood Ball-Mediated Transmission of Microbiome Members in the Dung Beetle, Onthophagus taurus (Coleoptera: Scarabaeidae). PLoS ONE.

[23] Xu L, Lu M, Xu D, Chen L, Sun J (2016). Sexual variation of bacterial microbiota of *Dendroctonus valens* guts and frass in relation to verbenone production. J Insect Physiol.

[24] Cheng D, Guo Z, Riegler M, Xi Z, Liang G, Xu Y. Gut symbiont enhances insecticide resistance in a significant pest, the oriental fruit fly *Bactrocera dorsalis* (Hendel). Microbiome. 2017;5(1):13. 10.1186/s40168-017-0236-z. 10.1186/s40168-017-0236-z PMC5286733 28143582

[25] Andongma AA, Wan L, Dong YC, Li P, Desneux N, White JA, et al. Pyrosequencing reveals a shift in symbiotic bacteria populations across life stages of *Bactrocera dorsalis*. Sci Rep. 2015;5. 10.1038/srep09470. 10.1038/srep09470 PMC5380164 25822599

[26] Saraithong P, Li Y, Saenphet K, Chen Z, Chantawannakul P (2017). Midgut bacterial communities in the giant Asian honeybee (*Apis dorsata*) across 4 developmental stages: a comparative study. Insect Sci.

[27] Caporaso JG, Lauber CL, Walters WA, Berglyons D, Huntley J, Fierer N (2012). Ultra-high-throughput microbial community analysis on the Illumina HiSeq and MiSeq platforms. Isme J.

[28] Fadrosh Douglas W, Ma Bing, Gajer Pawel, Sengamalay Naomi, Ott Sandra, Brotman Rebecca M, Ravel Jacques (2014). An improved dual-indexing approach for multiplexed 16S rRNA gene sequencing on the Illumina MiSeq platform. Microbiome.

[29] Magoč T, Salzberg SL (2011). FLASH: fast length adjustment of short reads to improve genome assemblies. Bioinformatics.

[30] Edgar RC (2013). UPARSE: highly accurate OTU sequences from microbial amplicon reads. Nat Methods.

[31] Caporaso JG, Kuczynski J, Stombaugh J, Bittinger K, Bushman FD, Costello EK (2010). QIIME allows analysis of high-throughput community sequencing data. Nat Methods.

[32] White JR, Nagarajan N, Pop M (2009). Statistical methods for detecting differentially abundant features in clinical metagenomic samples. PLoS Comput Biol.

[33] Wong CNA, Ng P, Douglas AE (2011). Low-diversity bacterial community in the gut of the fruitfly *Drosophila melanogaster*. Environ Microbiol.

[34] Yun Ji-Hyun, Roh Seong Woon, Whon Tae Woong, Jung Mi-Ja, Kim Min-Soo, Park Doo-Sang, Yoon Changmann, Nam Young-Do, Kim Yun-Ji, Choi Jung-Hye, Kim Joon-Yong, Shin Na-Ri, Kim Sung-Hee, Lee Won-Jae, Bae Jin-Woo (2014). Insect Gut Bacterial Diversity Determined by Environmental Habitat, Diet, Developmental Stage, and Phylogeny of Host. Applied and Environmental Microbiology.

[35] Ranjith MT, ManiChellappan, Harish ER, Girija D, Nazeem PA (2016). Bacterial communities associated with the gut of tomato fruit borer, *Helicoverpa armigera* (Hübner) (Lepidoptera: Noctuidae) based on Illumina next generation sequencing. J Asia Pac Entomol.

[36] Zurek Klara, Nayduch Dana (2016). Bacterial Associations Across House Fly Life History: Evidence for Transstadial Carriage From Managed Manure. Journal of Insect Science.

[37] Zajac BK, Sontigun N, Wannasan A, Verhoff MA, Sukontason K, Amendt J (2016). Application of DNA barcoding for identifying forensically relevant Diptera from northern Thailand. Parasitol Res.

[38] Gupta AK, Nayduch D, Verma P, Shah B, Ghate HV, Patole MS (2012). Phylogenetic characterization of bacteria in the gut of house flies (*Musca domestica* L.). FEMS Microbiol Ecol.

[39] Bahrndorff Simon, de Jonge Nadieh, Skovgård Henrik, Nielsen Jeppe Lund (2017). Bacterial Communities Associated with Houseflies (Musca domestica L.) Sampled within and between Farms. PLOS ONE.

[40] Su Zhijian, Zhang Minjing, Liu Xia, Tong Lei, Huang Yadong, Li Guanghong, Pang Yi (2010). Comparison of Bacterial Diversity in Wheat Bran and in the Gut of Larvae and Newly Emerged Adult of Musca domestica (Diptera: Muscidae) by Use of Ethidium Monoazide Reveals Bacterial Colonization. Journal of Economic Entomology.

[41] Zhao F, Stanley D, Wang Y, Zhu F, Lei CL (2009). Eicosanoids mediate nodulation reactions to a mollicute bacterium in larvae of the blowfly, *Chrysomya megacephala*. J Insect Physiol.

[42] Kohl KD, Amaya J, Passement CA, Dearing MD, McCue MD (2014). Unique and shared responses of the gut microbiota to prolonged fasting: a comparative study across five classes of vertebrate hosts. FEMS Microbiol Ecol.

[43] Oyaert M., De Baere T., Breyne J., De Laere E., Marien S., Waets P., Laffut W. (2013). First Case of Pseudoclavibacter bifida Bacteremia in an Immunocompromised Host with Chronic Obstructive Pulmonary Disease (COPD). Journal of Clinical Microbiology.

[44] Hwang Ok-Hwa, Raveendar Sebastian, Kim Young-Ju, Kim Ji-Hun, Kim Tae-Hun, Choi Dong-Yoon, Jeon Che Ok, Cho Sung-Back, Lee Kyung-Tai (2014). Deodorization of pig slurry and characterization of bacterial diversity using 16S rDNA sequence analysis. Journal of Microbiology.

[45] Albuquerque TA, Zurek L. Temporal changes in the bacterial community of animal feces and their correlation with stable fly oviposition, larval development, and adult fitness. Front Microbiol. 2014;5. 10.3389/fmicb.2014.00590. 10.3389/fmicb.2014.00590 PMC4226234 25426108

[46] Palm N, Dezoete M, Cullen T, Barry N, Stefanowski J, Hao L (2014). Immunoglobulin a coating identifies colitogenic bacteria in inflammatory bowel disease. Cell.

[47] Le BC, Gombert M, Robert S, Mercier E, Lanotte P (2015). Association of necrotizing wounds colonized by maggots with *Ignatzschineria*–associated septicemia. Emerg Infect Dis.

[48] Cao X-M, Chen T, Xu L-Z, Yao L-S, Qi J, Zhang X-L (2013). Complete genome sequence of *Wohlfahrtiimonas chitiniclastica* strain SH04, isolated from *Chrysomya megacephala* collected from Pudong international airport in China. Genome Announcements.

[49] Prévoteau A, Geirnaert A, Arends JB, Lannebère S, Van dWT, Rabaey K (2015). Hydrodynamic chronoamperometry for probing kinetics of anaerobic microbial metabolism-case study of *Faecalibacterium prausnitzii*. Sci Rep.

[50] Sokol H., Pigneur B., Watterlot L., Lakhdari O., Bermudez-Humaran L. G., Gratadoux J.-J., Blugeon S., Bridonneau C., Furet J.-P., Corthier G., Grangette C., Vasquez N., Pochart P., Trugnan G., Thomas G., Blottiere H. M., Dore J., Marteau P., Seksik P., Langella P. (2008). Faecalibacterium prausnitzii is an anti-inflammatory commensal bacterium identified by gut microbiota analysis of Crohn disease patients. Proceedings of the National Academy of Sciences.

[51] Serdani M, Curtis M, Miller ML, Kraus J, Putnam ML (2013). Loop-mediated isothermal amplification and polymerase chain reaction methods for specific and rapid detection of *Rhodococcus fascians*. Plant Dis.

