# Supplementary material for: The gut bacteria across life stages in the synanthropic fly Chrysomya megacephala
Source: BMC Microbiol. 2018 Oct 11;18:131. doi: 10.1186/s12866-018-1272-y (PMC6180576; doi:10.1186/s12866-018-1272-y)
Supplement: Supplementary file 1 — Table S1. Samples and their sequencing data processing. (DOCX 15 kb) [file 12866_2018_1272_MOESM1_ESM.docx]

**Table S1** Samples and their sequencing data processing

| **Sample name** | **Raw**  **reads** | **Clean**  **reads** | **Utilization Ratio of Reads (%)** | **Tags** | **OTUs** |
| --- | --- | --- | --- | --- | --- |
|  |  |  |  |  |  |
| Eggs.1 | 31819 | 31033 | 97.53 | 27700 | 134 |
| Eggs.2 | 32187 | 31270 | 97.15 | 28384 | 116 |
| Eggs.3 | 32424 | 31304 | 96.55 | 26738 | 161 |
| 1-d-Larvae.1 | 32286 | 31253 | 96.8 | 25937 | 340 |
| 1-d-Larvae.2 | 32303 | 31114 | 96.32 | 25330 | 337 |
| 1-d-Larvae.3 | 32473 | 31285 | 96.34 | 25370 | 320 |
| 5-d-Larvae.1 | 33006 | 31595 | 95.73 | 29284 | 254 |
| 5-d-Larvae.2 | 32900 | 31415 | 95.49 | 28630 | 305 |
| 5-d-Larvae.3 | 32966 | 31593 | 95.84 | 28002 | 330 |
| Pupae.1 | 32156 | 31058 | 96.59 | 29082 | 252 |
| Pupae.2 | 32325 | 30990 | 95.87 | 28885 | 177 |
| Pupae.3 | 32367 | 31244 | 96.53 | 29703 | 241 |
| Female.1 | 32414 | 31208 | 96.28 | 27695 | 202 |
| Female.2 | 32703 | 31564 | 96.52 | 28113 | 187 |
| Female.3 | 32690 | 31552 | 96.52 | 27856 | 185 |
| Male.1 | 32447 | 31203 | 96.17 | 27660 | 136 |
| Male.2 | 32321 | 31064 | 96.11 | 26647 | 126 |
| Male.3 | 32706 | 31500 | 96.31 | 27722 | 133 |
