# Supplementary material for: The gut bacteria across life stages in the synanthropic fly Chrysomya megacephala
Source: BMC Microbiol. 2018 Oct 11;18:131. doi: 10.1186/s12866-018-1272-y (PMC6180576; doi:10.1186/s12866-018-1272-y)
Supplement: Supplementary file 3 — Figure S1. Rarefaction curve based on OTUs. Mothur (v1.31.2) were used to calculate indices for rareaction curve based on observed species values. (DOCX 130 kb) [file 12866_2018_1272_MOESM3_ESM.docx]

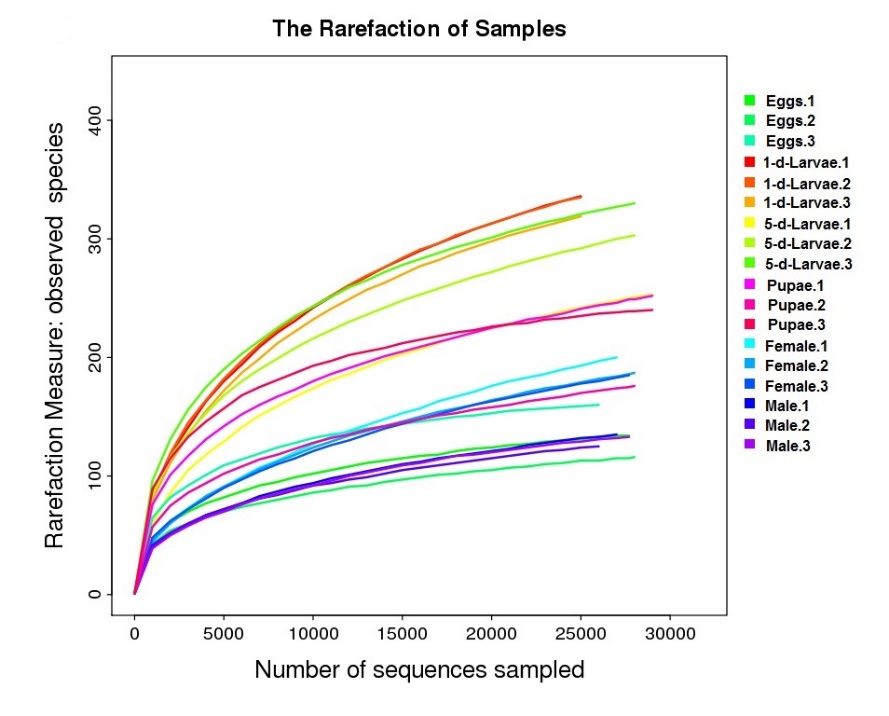


**Figure S1** Rarefaction curve based on OTUs. Mothur (v1.31.2) were used to calculate indices for rarefaction curve based on observed species values.
