# Supplementary material for: The gut bacteria across life stages in the synanthropic fly Chrysomya megacephala
Source: BMC Microbiol. 2018 Oct 11;18:131. doi: 10.1186/s12866-018-1272-y (PMC6180576; doi:10.1186/s12866-018-1272-y)
Supplement: Supplementary file 5 — Table S4. Comparative abundance of bacterial species between female and male of C. megacephala. (DOCX 17 kb) [file 12866_2018_1272_MOESM5_ESM.docx]

**Table S4 Comparative abundance of bacterial species between female and male of *C. megacephala***

| **Species** | **Female** | | **Male** | | | | **p-value** |
| --- | --- | --- | --- | --- | --- | --- | --- |
|  | **mean** | **SE** | | **mean** | **SE** |  | |
| *Acholeplasma laidlawii* | 0 | 0 | | 0.002405 | 0.002405 | 0.245089 | |
| *Akkermansia muciniphila* | 0 | 0 | | 0.003607 | 0.003607 | 0.345429 | |
| *Alcaligenes faecalis* | 0.008407 | 0.006708 | | 0.001251 | 0.001251 | 0.288857 | |
| *Arcobacter cryaerophilus* | 0 | 0 | | 0.013526 | 0.004616 | 0.055179 | |
| *Bacteroides coprosuis* | 0.003597 | 0.002073 | | 0.001251 | 0.001251 | 0.485429 | |
| *Blautia producta* | 0.003604 | 0.002085 | | 0.00241 | 0.00241 | 0.767679 | |
| *Brevundimonas diminuta* | 0.001204 | 0.001204 | | 0.001205 | 0.001205 | 1 | |
| *Clostridium butyricum* | 0.005993 | 0.00242 | | 0.012221 | 0.00439 | 0.215821 | |
| *Collinsella aerofaciens* | 0.005997 | 0.003169 | | 0 | 0 | 0.084357 | |
| *Commensalibacter intestini* | 1.05366 | 0.202348 | | 0.710737 | 0.160392 | 0.176786 | |
| *Corynebacterium variabile* | 0.112374 | 0.030019 | | 0.096214 | 0.01181 | 0.652179 | |
| *Desulfovibrio D168* | 0.001204 | 0.001204 | | 0 | 0 | 1 | |
| *Escherichia coli* | 0.021573 | 0.00723 | | 0.013302 | 0.011477 | 0.614143 | |
| *Eubacterium biforme* | 0.003604 | 0.002085 | | 0.001205 | 0.001205 | 0.438536 | |
| *Faecalibacterium prausnitzii* | 0.004783 | 0.001199 | | 0 | 0 | 0.03607* | |
| *Lactobacillus reuteri* | 0.152124 | 0.037408 | | 0.079569 | 0.013142 | 0.103 | |
| *Lactococcus garvieae* | 0.008344 | 0.004306 | | 0.002405 | 0.002405 | 0.2305 | |
| *Myroides odoratimimus* | 0.046745 | 0.029229 | | 0.086708 | 0.04814 | 0.560321 | |
| *Prevotella copri* | 0.043892 | 0.040356 | | 0 | 0 | 0.244929 | |
| *Prevotella stercorea* | 0.0083 | 0.0083 | | 0 | 0 | 0.345429 | |
| *Pseudoclavibacter bifida* | 0.001197 | 0.001197 | | 0 | 0 | 1 | |
| *Pseudomonas alcaligenes* | 0.009525 | 0.004257 | | 0.003615 | 0.003615 | 0.281571 | |
| *Pseudomonas viridiflava* | 0.002389 | 0.001195 | | 0.001251 | 0.001251 | 1 | |
| *Rhodococcus fascians* | 0 | 0 | | 0.006066 | 0.001157 | 0.01075* | |
| *Ruminococcus bromii* | 0 | 0 | | 0.003613 | 0.002087 | 0.1135 | |
| *Ruminococcus gnavus* | 0 | 0 | | 0.001205 | 0.001205 | 0.495066 | |
| *Ruminococcus torques* | 0.003597 | 0.002073 | | 0.001205 | 0.001205 | 0.427286 | |
| *Salinispora tropica* | 0.001186 | 0.001186 | | 0.003613 | 0.002087 | 0.313464 | |
| *Shewanella algae* | 0.025165 | 0.011074 | | 0.016898 | 0.006653 | 0.596857 | |
| *Sphingobacterium faecium* | 0.002393 | 0.002393 | | 0.003661 | 0.002088 | 0.7065 | |
| *Sphingobacterium multivorum* | 0.016654 | 0.009264 | | 0.002456 | 0.001229 | 0.138107 | |
| *Streptococcus luteciae*  Unclassified | 0.586757 | 0.126528 | | 0.447417 | 0.059912 | 0.449786 | |
| Unclassified | 97.86573 | 0.105504 | | 98.48098 | 0.242046 | 0.066607 | |

Note: Metastats (http://metastats.cbcb.umd.edu/) and R (v3.1.1) are used to determine which taxonomic groups were significantly different between groups of samples. We adjusted the obtained P-value by a Benjamini-Hochberg false discovery rate correction (function 'p. adjust' in the stats package of R(v3.1.1)). *indicate that significant difference was detected.
